# Supplementary material for: Content-rich biological network constructed by mining PubMed abstracts
Source: BMC Bioinformatics. 2004 Oct 8;5:147. doi: 10.1186/1471-2105-5-147 (PMC528731; doi:10.1186/1471-2105-5-147)
Supplement: Additional File 2 — The original results of the above study (non-essential files are deleted to keep the file size under the limit set by BMC bioinformatics). [file 1471-2105-5-147-S2.bz2 › chilibotAdditionalFile2/dip50/270ID2169352E1090/html/NR3C1_JUN.html]

 


 **NR3C1** and **JUN** 
  
Found 213 abstracts in PubMed, retrieved 50.  
 

 What does Google say? 
 PDF only 
| .edu only 

---

**Interactive relationship** (e.g. stimulation, inhibition, etc)

**Inhibitory relationship**- Overexpression of the human glucocorticoid receptor alpha  [ **NR3C1** ]  and beta isoforms inhibits AP 1  [ **JUN** ]  and NF kappaB activities hormone independently.  Ref: 12021843 J Mol Med, 2002
- Receptor Interacting Protein 140 Binds c  **Jun**  and Inhibits Estradiol Induced Activator Protein 1  [ **JUN** ]  Activity by Reversing Glucocorticoid Receptor  [ **NR3C1** ]  Interacting Protein 1 Effect.  Ref: 12554755 Mol Endocrinol, 2003
- The c  **Jun**  N terminal kinase JNK phosphorylates the glucocorticoid receptor  [ **NR3C1** ]  GR and inhibits GR mediated transcription.  Ref: 12351702 Mol Endocrinol, 2002
- Regardless of the AP 1  [ **JUN** ]  complex s composition, glucocorticoid receptor  [ **NR3C1** ]  activation inhibits AP 1  [ **JUN** ]  and prevents apoptosis.  Ref: 12766089 Invest Ophthalmol Vis Sci, 2003
**Neutral relationship**- We demonstrate here that glucocorticoid mediated inhibition of enhancer function involves glucocorticoid receptor  [ **NR3C1** ]  GR binding to the NF AT AP 1  [ **JUN** ]  sites.  Ref: 11509589 J Immunol, 2001
- Electrophoretic mobility shift assays showed that c  **Jun**  is unable to efficiently interact with the AP 1  [ **JUN** ]  like site present in the human glucocorticoid receptor  [ **NR3C1** ]  promoter.  Ref: 11325517 Mol Cell Endocrinol, 2001
- NIH3T3 cell clones overexpressing c  **Jun**  exhibited lower glucocorticoid receptor  [ **NR3C1** ]  mRNA levels, which suggests that the murine glucocorticoid receptor  [ **NR3C1** ]  gene can also be regulated by AP 1  [ **JUN** ] .  Ref: 11325517 Mol Cell Endocrinol, 2001
- Using this method it has been possible to dissociate goat uterine oestrogen receptor homodimers, heterodimers formed between the non activated oestrogen receptor naER and the oestrogen receptor activation factor E RAF of the goat uterus, c  **jun**  homodimers derived from bovine bone marrow and also glucocorticoid receptor  [ **NR3C1** ]  homodimers isolated from rat liver cytosol.  Ref: 11341964 Biochim Biophys Acta, 2001
- The glucocorticoid receptor  [ **NR3C1** ]  GR associated with col3A through protein protein interactions with AP 1  [ **JUN** ] , regardless of AP 1  [ **JUN** ]  subunit composition, and repressed transcription.  Ref: 11689447 EMBO J, 2001
- We have previously shown that induction is mediated by the direct interaction between c  **Jun**  and ERalpha, which stabilizes a multiprotein complex containing the coactivator GRIP1 glucocorticoid receptor  [ **NR3C1** ]  interacting protein 1.  Ref: 12554755 Mol Endocrinol, 2003
- Interleukin 2 IL 2, through the Janus kinase signal transducers and activators of transcription Jak STAT and mitogen activated protein kinase MAPK pathways, activates STAT5 and activated protein 1 AP 1  [ **JUN** ]  transcription factors, respectively, which are known to repress glucocorticoid receptor  [ **NR3C1** ]  GR activity, at least in part, through protein protein interactions.  Ref: 11435608 Mol Endocrinol, 2001
- Mammalian cell culture studies have shown that several members of the nuclear receptor super family such as glucocorticoid receptor  [ **NR3C1** ] , retinoic acid receptor and thyroid hormone receptor can repress the activity of AP 1  [ **JUN** ]  proteins by a mechanism that does not require the nuclear receptor to bind to DNA directly, but that is otherwise poorly understood.  Ref: 12049770 Mech Dev, 2002
- Consistently with previous findings, a glucocorticoid receptor  [ **NR3C1** ]  mutant GRdim, which is deficient in dimerization, DNA binding, and transactivation, but retains AP 1  [ **JUN** ]  transrepressing activity, was as efficient as wild type GR in mediating the same effects of dexamethasone on JNK in transfected Cos 7 cells.  Ref: 10974006 J Cell Biol, 2000
- Nuclear export of glucocorticoid receptor  [ **NR3C1** ]  is enhanced by c  **Jun**  N terminal kinase mediated phosphorylation.  Ref: 12351702 Mol Endocrinol, 2002
- Glucocorticoids inhibit cytokine induced transcription factors NFkB, AP1  [ **JUN** ]  and cytokines enhance glucocorticoid receptor  [ **NR3C1** ]  GR transcriptional activity, thus reciprocally fine tuning immunological control mechanisms.  Ref: 10969177 J Neuroimmunol, 2000

**Non-interactive relationship** (e.g. studied together, co-existance, homology, etc.)

- Both factors make use of the coactivator cAMP response element binding protein CREB binding protein CBP to enhance their transcriptional activities, which led to the hypothesis that a mutual antagonism between p65 or c  **Jun**  and activated glucocorticoid receptor  [ **NR3C1** ]  GR results from a limited amount of CBP.  Ref: 11158329 Mol Endocrinol, 2001
- Antagonism of glucocorticoid receptor  [ **NR3C1** ]  transactivity and cell growth inhibition by transforming growth factor beta through AP 1  [ **JUN** ]  mediated transcriptional repression.  Ref: 12379279 Int J Biochem Cell Biol, 2002
- Interleukin 2 inhibits glucocorticoid receptor  [ **NR3C1** ]  transcriptional activity through a mechanism involving STAT5 signal transducer and activator of transcription 5 but not AP 1  [ **JUN** ] .  Ref: 11435608 Mol Endocrinol, 2001
- Glucocorticoid receptor  [ **NR3C1** ]  GR and activator protein AP 1  [ **JUN** ]  activities were shown by Western blot analysis and electrophoretic mobility shift assay EMSA of retinal nuclear extracts.  Ref: 11381074 Invest Ophthalmol Vis Sci, 2001
- The immunosuppressive effects of glucocorticoids arise largely by inhibition of cytokine gene expression, which has been ascribed to interference between the glucocorticoid receptor  [ **NR3C1** ]  and transcription factors such as AP 1  [ **JUN** ]  and NF kappa B as well as by competition for common coactivators.  Ref: 11397794 J Biol Chem, 2001
- Suppression of granulocyte macrophage colony stimulating factor expression by glucocorticoids involves inhibition of enhancer function by the glucocorticoid receptor  [ **NR3C1** ]  binding to composite NF AT activator protein 1  [ **JUN** ]  elements.  Ref: 11509589 J Immunol, 2001
- Glucocorticoids GCs function, in part, through the ability of the glucocorticoid receptor  [ **NR3C1** ]  GR to activate gene expression and in part through the transrepression of AP 1  [ **JUN** ]  and NF kappaB.  Ref: 11106637 J Biol Chem, 2001
- These results provide a new mechanism for cross talk between the glucocorticoid receptor  [ **NR3C1** ]  and the AP 1  [ **JUN** ]  family of transcription factors in the absence of glucocorticoid ligands.  Ref: 11325517 Mol Cell Endocrinol, 2001
- Repression of glucocorticoid receptor  [ **NR3C1** ]  gene transcription by c  **Jun** .  Ref: 11325517 Mol Cell Endocrinol, 2001
- RU486, a glucocorticoid receptor  [ **NR3C1** ]  antagonist, reversed MP inhibition of AP 1  [ **JUN** ]  activation.  Ref: 11150324 J Neurosci, 2001
- AP 1  [ **JUN** ]  glucocorticoid receptor  [ **NR3C1** ]  crosstalk taken to a higher level.  Ref: 11375114 J Endocrinol, 2001
- Importantly, AP 1  [ **JUN** ]  responsiveness for glucocorticoid receptor  [ **NR3C1** ]  mediated inhibition was preserved in Fos Fosl1 Fosl1 mice.  Ref: 11953459 J Neurochem, 2002
- Expression of c fos, rather than c  **jun**  or glucocorticoid receptor  [ **NR3C1** ]  mRNA, correlates with decreased glucocorticoid response of peripheral blood mononuclear cells in asthma.  Ref: 12400872 Int Immunopharmacol, 2002
- The ligand activated glucocorticoid receptor  [ **NR3C1** ]  inhibits activity of the AP 1  [ **JUN** ]  transcription factor and the purpose of this study was to test the hypothesis that up regulation or overexpression of AP1  [ **JUN** ]  binding activity may be an important mechanism of glucocorticoid resistance in ALL and CLL.  Ref: 11237062 Leukemia, 2001
- The regulation of glucocorticoid receptor  [ **NR3C1** ]  gene expression by members of the AP 1  [ **JUN** ]  family was examined in glucocorticoid free NIH3T3 cells transfected with the human glucocorticoid receptor  [ **NR3C1** ]  gene promoter driving expression of a CAT reporter gene.  Ref: 11325517 Mol Cell Endocrinol, 2001
- This promoter region contains recognition consensus sequences for various transcription factors, including one activation protein 1 AP 1  [ **JUN** ]  site, two cyclic AMP responsive element sites, one estrogen receptor element site and four glucocorticoid receptor  [ **NR3C1** ]  element sites.  Ref: 11406293 Brain Res Mol Brain Res, 2001
- Cross talk between glucocorticoid receptor  [ **NR3C1** ]  and AP 1  [ **JUN** ] .  Ref: 11402341 Oncogene, 2001
- General GC responsiveness was not compromised, as evidenced by Dex mediated suppression of endogenous c Myc and cyclin D3, and induction of c  **Jun**  and the glucocorticoid receptor  [ **NR3C1** ] .  Ref: 11498786 Oncogene, 2001
- The elements likely for nuclear receptors retinoic acid receptor, retinoic acid X receptor, hepatocyte nuclear factor 3, glucocorticoid receptor  [ **NR3C1** ] , nuclear factor kappaB and activator protein 1  [ **JUN** ]  were also found.  Ref: 12384299 Gene, 2002
- These results suggest that AP 1  [ **JUN** ]  proteins and glucocorticoid receptor  [ **NR3C1** ]  regulate transcription of the GnRH R promoter in a heterologous system.  Ref: 12644302 Mol Cell Endocrinol, 2003
- Concomitantly, the expression of some selected endogenous genes, such as those coding for c  **Jun**  and the glucocorticoid receptor  [ **NR3C1** ] , was enhanced significantly relative to actin.  Ref: 11329370 J Cell Sci, 2001
- Employing DNaseI protection and electrophoretic mobility shift assays EMSA, we now demonstrate that DNA transcription factor protein complexes are formed on the c fms first promoter at a composite regulatory element containing overlapping binding sites for AP 1  [ **JUN** ]  proteins, bHLH factors, and the glucocorticoid receptor  [ **NR3C1** ]  GR.  Ref: 11891846 J Cell Biochem, 2002
- Cross talk between different transcription factors, notably between the glucocorticoid receptor  [ **NR3C1** ]  and AP 1  [ **JUN** ] , has been discovered more than 10 years ago a bona fide transcription factor, without apparent need for its own direct DNA contact, influences the activity of another transcription factor.  Ref: 11402341 Oncogene, 2001
